# Clinical characteristics and burden of illness in patients with hereditary angioedema: findings from a multinational patient survey

**DOI:** 10.1186/s13023-021-01717-4

**Published:** 2021-02-18

**Authors:** Joan Mendivil, Ryan Murphy, Marie de la Cruz, Ellen Janssen, Henrik Balle Boysen, Gagan Jain, Emel Aygören-Pürsün, Ishan Hirji, Giovanna Devercelli

**Affiliations:** 1 Takeda Pharmaceuticals International AG, Thurgauerstrasse 130, 8152 Glattpark-Opfikon, Zurich, Switzerland; 2 ICON, Gaithersburg, MD USA; 3 HAE International (HAEi), Horsens, Denmark; 4 Takeda Pharmaceutical Company Limited, Lexington, MA USA; 5 Department for Children and Adolescents, Angioedema Centre, University Hospital Frankfurt, Goethe University, Frankfurt, Germany

**Keywords:** Hereditary angioedema, Burden of illness, Quality of life, Long-term prophylaxis, Surveys and questionnaires, Cross-sectional studies

## Abstract

**Background:**

Hereditary angioedema (HAE) is a rare, debilitating, genetic disease characterized by unpredictable, recurrent, and potentially fatal swelling of the skin and mucous membranes. We conducted a noninterventional, cross-sectional, web-based survey of patients with a self-reported diagnosis of HAE type 1/2 in Australia, Austria, Canada, France, Germany, Spain, Switzerland, and the United Kingdom to gain a comprehensive real-world understanding of the characteristics of HAE and its burden from the perspective of the patient. The survey included questions on clinical and demographic characteristics, burden of disease, and treatment. Instruments used to measure patient-reported outcomes included the Angioedema Quality of Life questionnaire (AE-QoL), 12-Item Short-Form Health Survey (SF-12v2), Angioedema Control Test (AECT), Hospital Anxiety and Depression Scale (HADS), and Work Productivity and Impairment questionnaire (WPAI). Data were analyzed with descriptive statistics.

**Results:**

A total of 242 patients (67.4% female; mean [range] age 43.8 [18–92] years) completed the survey. The mean (SD) age at first symptoms was 11.5 (8.9) years, while diagnosis occurred at 20.8 (13.2) years. Patients reported a mean (SD) of 12.5 (14.1) attacks in the past 6 months. The most recent attack occurred within the past month in 79.7% of patients; most were of moderate severity, 6.6% affected the larynx, 21.9% lasted ≥ 3 days, and 76.4% were treated with on-demand medication. Hospitalizations and emergency/urgent care visits were highest for patients with more attacks. At the time of the survey, 62.4% of patients were using long-term prophylaxis, including 34.4% using androgens. Moderate to severe anxiety and depression were reported in 38.0% and 17.4% of patients, respectively, as measured using the HADS. The severity of anxiety and depression was associated with poorer quality of life and productivity, measured using the AECT (mean overall score 8.00 [moderate perceived disease control]), AE-QoL, WPAI, and SF-12v2. Scores for AECT, AE-QoL, and WPAI were also worse with a higher number of attacks.

**Conclusions:**

This survey study of a broad international sample of patients with HAE showed that despite the availability of on-demand treatment and long-term prophylaxis for the prevention of attacks, patients across a wide geographical area continue to have high disease activity, likely due to restrictions in the availability of medications or incorrect use. Subsequently, significant disease burden, including impaired quality of life and mental health and decreased productivity, was evident. Increased patient education and access to newer, more effective therapies are needed.

## Background

Hereditary angioedema (HAE) is a rare genetic disease affecting 1.5 in 100,000 people [1]. It results from a deficiency in the level or function of the C1 inhibitor (C1-INH) protein (types 1 and 2, respectively) [2], and is characterized by debilitating attacks of angioedema affecting the skin and mucous membranes. Attacks most commonly affect the extremities, face, abdomen, and larynx; laryngeal attacks are of particular concern, as they are potentially fatal [3].

HAE attacks recur throughout life with unpredictable frequency and severity, and most last several days if untreated [3]. As such, HAE has an extensive short- and long-term impact on patients’ lives [4]. Daily function may be impaired, resulting in reduced productivity or lost days at work or school, which negatively affects advancement and overall well-being [5–7]. Depression and anxiety were reported in a large proportion of patients with HAE [6]. Fear of future attacks often limits patients’ ability to socialize or travel, and many patients have concerns about their children inheriting HAE [8]. In addition, there is a heavy burden for the families and caregivers of patients [7, 8].

At the time of this study, treatments for HAE included on-demand medications to treat attacks after onset (such as icatibant, C1-INH inhibitor [Berinert® and Cinryze®], and ecallantide), and long- or short-term prophylactic therapy to prevent attacks (such as Cinryze®, androgens, and antifibrinolytics) [9]. However, despite improvements in the available HAE therapies, unmet needs remained; treatments require frequent dosing or intravenous administration, have limited efficacy, or are associated with poor tolerability [10].

While there has been increased interest in trying to better understand the burden of illness in HAE in recent years, key gaps in the literature remain, in particular a comprehensive, real-world understanding of the variability of HAE, its human and economic burdens, and its impact on quality of life directly from the patient’s perspective. A survey was thus conducted in Australia, Canada, and 6 European countries from July to October 2018 to gain this information. The findings from the survey are described herein and may help inform decision-making so that patients’ desired outcomes can be realized.

## Results

### Patient demographics and clinical history

A total of 617 patients were screened; of these, 371 did not meet the eligibility criteria and 4 did not consent to participate in the survey. Most screen failures were due to patients having “other” types of HAE (92/207 screen failures; 44.4%) or “did not know” what type of HAE they have (115/207; 55.6%) (see Additional File 1: Figure S1). A total of 242/617 (39.2%) patients across all countries met all the inclusion criteria, provided informed consent, and completed the survey. Patients were 18–92 years of age (mean [SD] 43.8 [14.7]). Most were from France (24.0%) and the United Kingdom (23.6%), female (67.4%), had HAE type 1 (81.8%), and had a family history of HAE (79.3%) (Table 1). Patients spent an average of 44.0 min completing the survey.

**Table 1:** Demographic and clinical characteristics of patients (*N* = 242)

Parameter	Value
Country, *n* (%)	
France	58 (24.0)
United Kingdom	57 (23.6)
Spain	39 (16.1)
Canada	32 (13.2)
Australia	28 (11.6)
Switzerland	8 (3.3)
Germany	7 (2.9)
Austria	13 (5.4)
Age, years	
Mean (SD)	43.8 (14.7)
Range	18–92
Sex, *n* (%)	
Female	163 (67.4)
Male	78 (32.2)
Not indicated	1 (0.4)
HAE type, *n* (%)	
Type 1	198 (81.8)
Type 2	44 (18.2)
Mean (SD) age at onset of first HAE symptoms, years	11.5 (8.9)
Mean (SD) age at HAE diagnosis, years	20.8 (13.2)
Mean (SD) delay in diagnosis,^a^ years	9.3 (11.0)
Family history of HAE, *n* (%)	
Yes	192 (79.3)
No	45 (18.6)
Not sure	5 (2.1)
Comorbidities,^b^ *n* (%)	
Anxiety	63 (26.0)
Gastrointestinal disorder	43 (17.8)
Depression	41 (16.9)
High cholesterol	33 (13.6)
Hypertension	31 (12.8)

*HAE* hereditary angioedema

^a^Delay in diagnosis was calculated as the difference between age at diagnosis and age at onset of first HAE symptoms

^b^Comorbidities reported by ≥ 10% of patients are listed; multiple comorbidities could be reported by the same patient

The mean (range) age at onset of first HAE symptoms was 11.5 (0–58) years, while the mean (range) age at HAE diagnosis was 20.8 (0–59) years. The age at diagnosis was similar across countries (see Additional File 1: Table S1). Mean ages at symptom onset and diagnosis were slightly earlier for patients with HAE type 1 (10.8 and 20.1 years, respectively) than for patients with HAE type 2 (15.0 and 24.1 years, respectively).

The most commonly reported comorbidities were anxiety (26.0%), gastrointestinal disorders (17.8%), depression (16.9%), high cholesterol (13.6%), and hypertension (12.8%) (see Additional File 1: Figure S2). Among patients with anxiety, 33.3% reported using medication to treat their anxiety, and among patients with depression, 43.9% reported using medication to treat their depression.

### HAE attack characteristics, symptoms, and severity

**Fig. 1:**
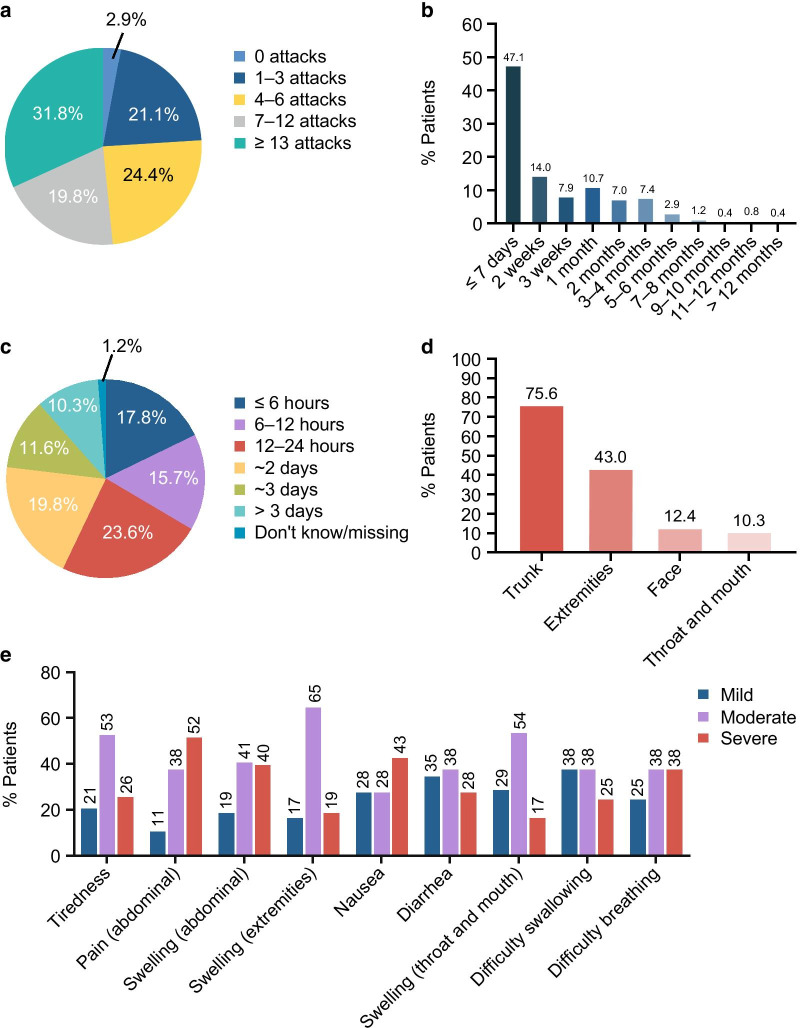
Characteristics of attacks. **a** Number of attacks in the past 6 months (*n* = 242). **b** Time since the most recent attack (*n* = 242). Patients were asked “When was your most recent angioedema attack? Please select the answer closest to the time when your first symptom of the attack appeared.” **c** Duration of the most recent attack (*n* = 242). Patients were asked “What was the duration of your most recent angioedema attack? Consider the time from when your first symptom appeared to when you noticed that all of the symptoms of the attack had disappeared.” **d** Location of the most recent attack (*n* = 242). More than 1 location could have been affected. Patients were asked “What area(s) of your body was (were) affected in your most recent angioedema attack? Select all that apply.” **e** Symptoms and symptom severity during the most recent attack (*n* = 242). Patients could select more than one response. Patients were asked “Which of the following symptoms, if any, did you experience as part of your most recent angioedema attack? Select all that apply.” and “Thinking of your most recent angioedema attack AT ITS WORST, please rate each symptom’s severity as None, Mild, Moderate, or Severe.”

Patients reported the occurrence of a mean (range) of 12.5 (0–90) attacks in the previous 6 months; 31.8% of patients had ≥ 13 attacks in the past 6 months, and 19.8% had 7–12 attacks (Fig. 1a). The mean (SD) number of attacks was lowest among patients in France (7.10 [8.88]) and highest among patients in Switzerland (18.38 [19.00]) (see Additional File 1: Table S2). Attacks were more frequent among patients with HAE type 1 than those with HAE type 2 (mean [SD] 13.19 [14.96] vs. 9.43 [8.83] attacks in 6 months, respectively).

In 79.7% of patients, the most recent attack occurred within the past month; furthermore, in 47.1% of patients, it occurred within the last 7 days (Fig. 1b). Only 2.8% of patients reported experiencing their most recent attack ≥ 6 months ago. In describing their most recent attack, 57.1% of patients reported that it lasted ≤ 24 h, while 21.9% experienced an attack that lasted ≥ 3 days (Fig. 1c). Attacks most frequently affected the trunk (75.6%) and the extremities (43.0%) (Fig. 1d). Attacks affecting the larynx were reported in 6.6% of patients. Half of patients (50.0%) reported that more than 1 location on their body was affected in their most recent attack. The most commonly reported symptoms were tiredness, abdominal pain, and abdominal swelling, each of which was reported by more than 50% of patients. Swelling in the extremities, nausea, diarrhea, dizziness, and pain in locations other than the abdomen were also reported in 20.2% to 38% of patients. Life-threatening symptoms included swelling in the throat or mouth (9.9%), difficulty swallowing (7%), and difficulty breathing (7%). Most symptoms were considered to be of moderate severity. However, abdominal pain and nausea were considered severe by 52% and 43% of patients, respectively (Fig. 1e).

### Therapeutic management of HAE

A total of 185/242 patients (76.4%) used on-demand medication to treat their most recent attack; among them, plasma-derived C1-INH products were the most commonly used (55.1%), followed by icatibant (51.4%). Twenty-four patients (9.9%) used both C1-INH and icatibant to treat attacks. Other medications (0.5–5.4%) included tranexamic acid, pain medications, intravenous fluids, antiemetics, anxiety/sleep medication, corticosteroids, and recombinant C1-INH. The most common reason for not using on-demand medication was that the attack was minor or mild (78/242; 32.2%); other reasons included a limited supply of medication (9.1%), cost of medication (4.6%), advised not to by physician (9.5%), it was not stated in the individual action plan from physician (0.8%), and other (2.1%).

**Fig. 2:**
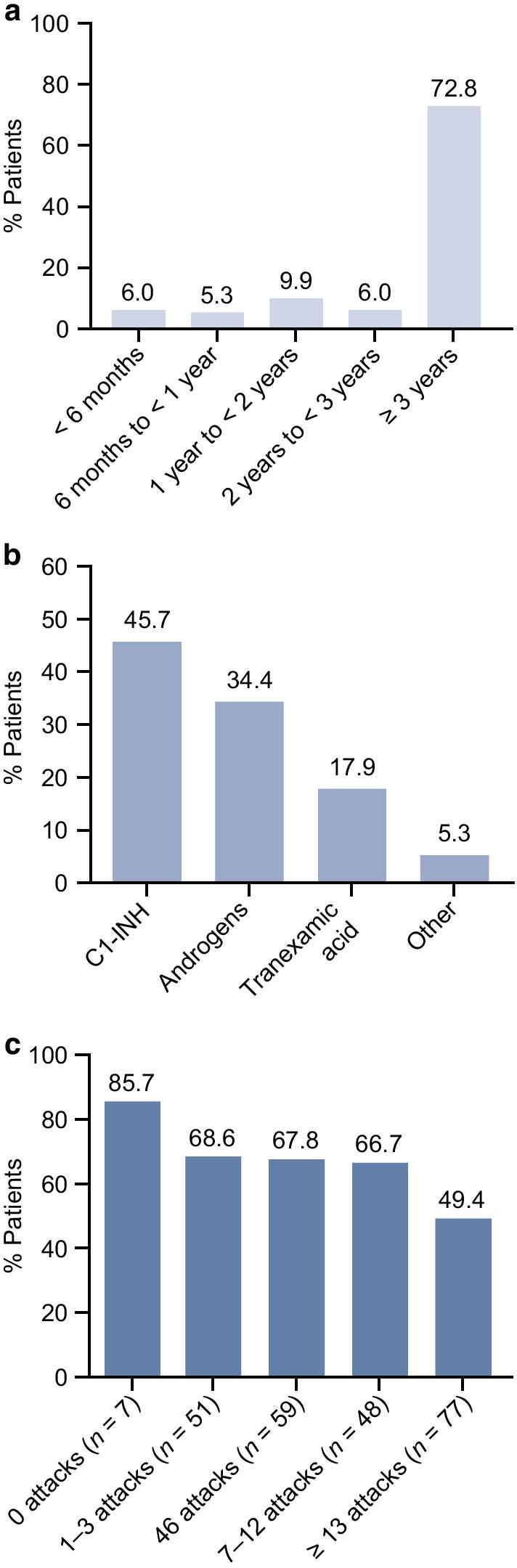
Current use of LTP. **a** Duration of current LTP use (among *n* = 151 patients currently using LTP). Patients were asked “How long have you been taking a medication on a long-term (regular, ongoing) basis TO PREVENT angioedema attacks from happening?” **b** Type of LTP currently used (*n* = 151). Patients may have chosen more than one. Patients were asked “Which of the following medications do you currently take on a long-term (regular, ongoing) basis TO PREVENT angioedema attacks from happening? Select all that apply.” C1-INH includes Berinert® and Cinryze®; androgens include danazol, Winstrol®; and Oxandrin®; and tranexamic acid includes tranexamic acid and Lysteda®. Patients were able to enter free text under “other”; the most frequently listed medications included Firazyr® and Exacyl®. **c** Proportion of patients currently using LTP stratified by number of attacks in the last 6 months. *C1-INH* C1 inhibitor, *LTP* long-term prophylaxis

A total of 151/242 (62.4%) patients reported that they were currently using long-term prophylaxis (LTP); of these, 72.8% had been on LTP for ≥ 3 years, and 88.7% had been on LTP for ≥ 1 year (Fig. 2a). Patients in Australia had the highest rate of LTP use (78.6%), while patients in Germany had the lowest (14.3%), although the sample size from Germany was small (see Additional File 1: Figure S3). The most commonly used LTP treatment was C1-INH (45.7% of patients who were currently on LTP, including 32.5% who used Berinert® and 14.6% who used Cinryze®) (Fig. 2b). Androgens were used by 34.4% of patients overall, including ~ 40% of patients in Australia, France, and Spain (Table 2). Most patients using C1-INH for LTP reported taking their medication every 3–4 days, while most patients using androgens took their medication every day.

**Table 2:** Current LTP and STP treatment by country

Treatment,^a^ *n* (%)	AU (*n* = 28)	AT (*n* = 13)	CA (*n* = 32)	FR (*n* = 58)	DE (*n* = 7)	ES (*n* = 39)	CH (*n* = 8)	UK (*n* = 57)
**LTP**	***n* = 22**	***n* = 2**	***n* = 23**	***n* = 35**	***n* = 1**	***n* = 28**	***n* = 2**	***n* = 38**
Cinryze® (C1 esterase inhibitor [human])	–	1 (50.0)	4 (17.4)	7 (20.0)	1 (100.0)	1 (3.6)	–	8 (21.1)
Berinert® (C1 esterase inhibitor [human])	10 (45.5)	–	16 (69.6)	2 (5.7)	–	7 (25.0)	1 (50.0)	13 (34.2)
Lysteda® (tranexamic acid)	–	–	3 (13.0)	2 (5.7)	–	2 (7.1)	–	12 (31.6)
Tranexamic acid	8 (36.4)	–	–	–	–	–	–	–
Azol®/danazol	9 (40.9)	–	1 (4.3)	15 (42.9)	–	11 (39.3)	–	7 (18.4)
Winstrol® (stanozolol)	–	–	–	–	–	10 (35.7)	–	1 (2.6)
Oxandrin® (oxandrolone)	–	–	–	–	–	–	–	1 (2.6)
Other	–	1 (50.0)	3 (13.0)	15 (42.9)	–	1 (3.6)	1 (50.0)	4 (10.5)
**STP**	***n* = 18**	***n* = 8**	***n* = 21**	***n* = 30**^b^	***n* = 6**	***n* = 30**	***n* = 4**	***n* = 39**
Cinryze® (C1 esterase inhibitor [human])	1 (5.6)	2 (25.0)	2 (9.5)	4 (13.3)	1 (16.7)	5 (16.7)	–	7 (17.9)
Berinert® (C1 esterase inhibitor [human])	13 (72.2)	6 (75.0)	18 (85.7)	11 (36.7)	5 (83.3)	19 (63.3)	4 (100.0)	28 (71.8)
Lysteda® (tranexamic acid)	–	–	2 (9.5)	1 (3.3)	–	1 (3.3)	–	3 (7.7)
Tranexamic acid	1 (5.6)	–	–	–	–	–	–	–
Azol®/danazol	6 (33.3)	–	1 (4.8)	13 (43.3)	–	8 (26.7)	–	3 (7.7)
Winstrol® (stanozolol)	–	–	–	–	–	4 (13.3)	–	–
Other	2 (11.1)	–	–	–	–	–	–	–

*AU* Australia, *AT* Austria, *CA* Canada, *CH* Switzerland, *DE* Germany, *ES* Spain, *FR* France, *LTP* long-term prophylaxis, *STP* short-term prophylaxis, *UK* United Kingdom

^a^Patients could select > 1 treatment

^b^Missing data for 1 patient

When stratified by the number of attacks in the past 6 months, LTP use was highest (85.7%) among patients who had no attacks, and lowest (49.4%) among patients with ≥ 13 attacks (Fig. 2c). Among 58 patients with few attacks (0 or 1–3) in the past 6 months, 8 (13.8%) reported past LTP use; the most common reasons for discontinuation were “side effects” (50%), “attacks not frequent enough” (37.5%), “didn’t like to take medication over a long period” (25%), and “prefer to take an on-demand treatment” (25%). In comparison, past LTP use was reported by 33/125 patients (26.4%) with ≥ 7 attacks in the past 6 months; “side effects” (42.4%), “medication not working as expected” (39.4%), and “health care provider recommendation” (30.3%) were the most frequent reasons for discontinuation.

Short-term prophylaxis (STP) had been used by 64.5% of patients overall, and was most common among those who had experienced ≥ 13 attacks in the past 6 months (77.9%). STP use was also reported by 49.2% of patients who had not experienced an attack in the past 6 months. The most commonly used medications for STP were C1-INH (87.2%) and androgens (22.4%). Use of STP ranged from 50.0% to 85.7% of patients across countries (see Additional File 1: Figure S4), and Berinert® was the most commonly reported medication for STP in each country except France, where Azol®/danazol was the most common (Table 2).

Use of LTP and STP was comparable between patients according to HAE type. LTP was used by 61.6% and 65.9% of patients with HAE type 1 and 2, respectively. STP was used by 65.2% and 61.4% of patients with HAE type 1 and 2, respectively. Some patients used LTP and STP concurrently.

### Health care resource utilization

Almost all patients (97.1%) reported regularly seeing a medical professional for HAE during the past year. The majority of patients across all countries were treated by an allergist/immunologist for HAE, except in Austria, France, and Germany, where the majority of patients were treated by a dermatologist, general practitioner/internist, and hematologist, respectively (see Additional File 1: Table S3). Regarding HAE-related issues, most patients (64.5%) were seeing an allergist/immunologist an average of 2.8 times per year, while 9.5% were seeing a physician assistant/nurse practitioner 22.5 times per year. Among patients who saw an allergist/immunologist for the management of HAE, the number of visits in the past year was highest (66%) in patients who experienced ≥ 7 attacks in the past 6 months. Among patients who saw a general practitioner/internist, the number of visits was highest among those with fewer attacks; 42.9% of patients with no attacks and 49.0% with 1–3 attacks in the past 6 months visited a general practitioner/internist in the last year.

During the past year, 37.6%, 19.4%, and 18.2% of patients reported ≥ 1 HAE-related emergency room visit, hospitalization, or urgent care visit, respectively. These visits were most frequent for patients with more attacks in the last 6 months. Patients in Austria had the highest number of visits to the emergency room and the highest number of hospitalizations (see Additional File 1: Table S4).

### Burden of HAE

#### General health status

The 12-Item Short-Form Health Survey (SF-12v2) [11, 12] was used to assess physical and mental health over the past week. Overall physical and mental health composite scores were 49.26 (9.30) and 43.09 (11.23), respectively, indicating poorer mental health in patients with HAE compared with the general population. Mental health composite scores were less than 50 for patients across all countries (see Additional File 1: Table S5). Composite scores for physical and mental health remained consistent across attack frequencies (see Additional File 1: Figure S5), but declined with higher levels of anxiety and depression, as measured using the Hospital Anxiety and Depression Scale (HADS) [13, 14]. The mental health composite score was affected to a greater extent by anxiety and depression, as measured using the HADS, than the physical health composite score.

#### Anxiety and depression

**Fig. 3:**
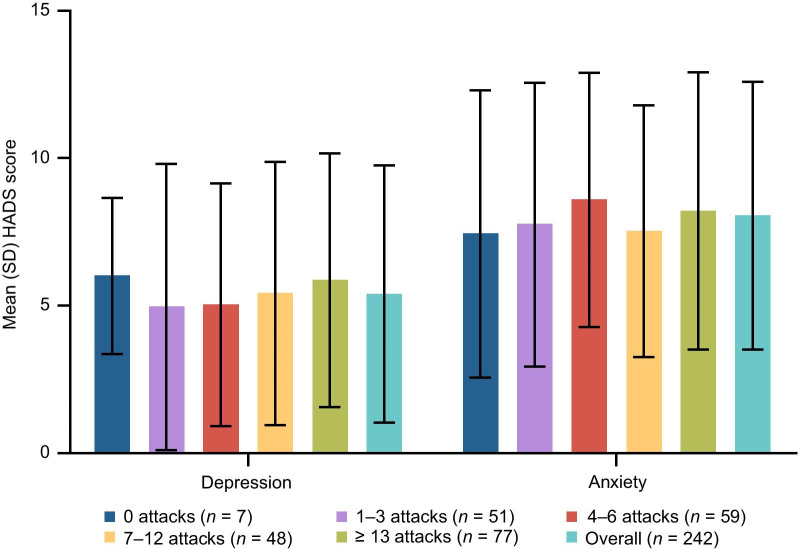
Mean HADS scores for the depression and anxiety subscales. Scores are shown by the number of HAE attacks in the previous 6 months. *HADS* Hospital Anxiety and Depression Scale, *HAE* hereditary angioedema

Patients’ anxiety and depression over the past week were assessed using the HADS [13, 14]. The mean (SD) total HADS score for all patients was 13.43 (8.17), indicating a moderate level of general psychological distress. Mean total scores were greater than 7 across all countries (see Additional File 1: Table S6). Moderate to severe anxiety and depression were reported in 38.0% and 17.4% of patients, respectively. Overall, scores for anxiety were higher than for depression, and the number of attacks in the previous 6 months did not affect the severity of either subscale (Fig. 3).

#### Control of disease

Angioedema-related disease control over the last 3 months was assessed using the Angioedema Control Test (AECT) [15, 16]. Overall, regarding the last 3 months, 45.0% of patients reported experiencing angioedema “often” or “very often”; 26.9% reported experiencing “much” or “very much” impairment in quality of life due to angioedema; 44.3% reported being “much” or “very much” bothered by the unpredictability of their angioedema; and 57.0% reported that their angioedema was “often” or “very often” controlled by their current treatment.

**Fig. 4:**
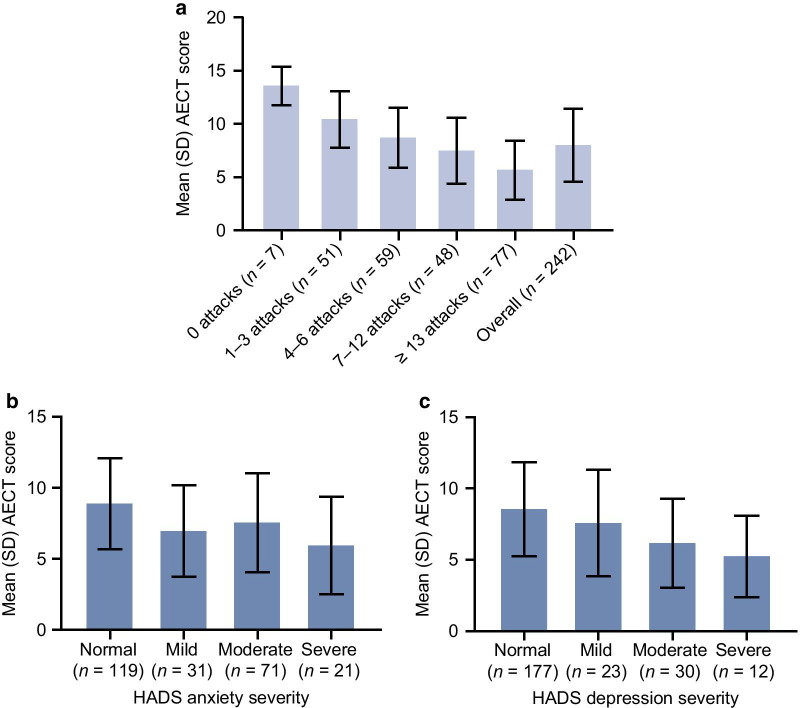
Mean AECT scores. Scores are shown by number of HAE attacks in the previous 6 months (**a**) and by HADS anxiety (**b**) and depression (**c**) subscales. *AECT* Angioedema Control Test, *HADS* Hospital Anxiety and Depression Scale, *HAE* hereditary angioedema

The mean (SD) AECT score overall was 8.00 (3.44), indicating poor disease control [16]. Mean scores ranged from 6.23 for patients in Austria to 9.69 for patients in France (see Additional File 1: Table S7). The majority of patients (81.8%) had a score less than 10. AECT scores decreased as the number of attacks increased; scores were 13.57 (1.81) for patients with no attacks in the past 6 months, and 5.68 (2.77) for patients with ≥ 13 attacks (Fig. 4a). Half of patients (50.0%) who had 1–3 attacks in the past 6 months reported controlled angioedema (see Additional File 1: Figure S6). Scores were also lower in patients who reported more severe anxiety and depression on the HADS. AECT scores decreased from 8.90 (3.21) in patients with a normal level of anxiety to 5.95 (3.44) in patients with severe anxiety (Fig. 4b), and scores decreased from 8.55 (3.30) in patients categorized as “normal” to 5.25 (2.86) in patients with severe depression (Fig. 4c).

#### Quality of life

**Fig. 5:**
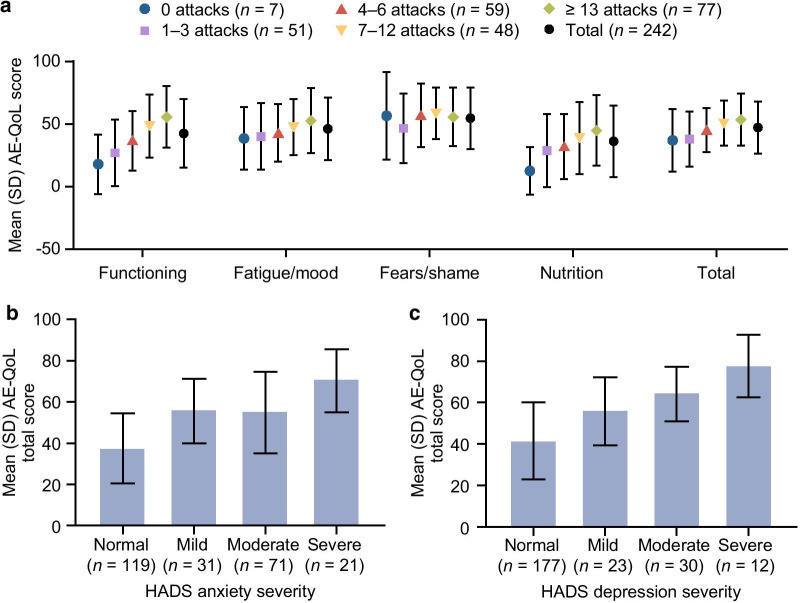
Mean AE-QoL scores. Scores are shown by number of HAE attacks in the previous 6 months (**a**) and by HADS anxiety (**b**) and depression (**c**) subscales. *AE-QoL* Angioedema Quality of Life questionnaire, *HADS* Hospital Anxiety and Depression Scale, *HAE* hereditary angioedema

The Angioedema Quality of Life questionnaire (AE-QoL) [17] was used to assess patients’ experiences with angioedema attacks over the past 4 weeks. The mean (SD) AE-QoL total score was 47.14 (20.69), indicating a moderate level of impairment. Scores were highest for the fears/shame domain (mean [SD] 54.68 [24.67]), followed by fatigue/mood (46.24 [24.95]), functioning (42.46 [27.39]), and nutrition (36.16 [28.62]). Total scores ranged from 27.52 for patients in Germany (no impairment) to 58.56 for patients in Spain (severe impairment) (see Additional File 1: Table S8). Total scores were generally higher with a higher number of reported attacks (Fig. 5a), ranging from 36.97 (25.01), or mild impairment, among patients who reported no attacks in the past 6 months to 53.55 (20.73), or severe impairment, for patients who reported ≥ 13 attacks in the past 6 months. Scores for the functioning, fatigue/mood, and nutrition domains increased with the number of attacks, but fears/shame scores remained similar regardless of the number of attacks. In addition, scores across all AE-QoL domains increased with the severity of anxiety (Fig. 5b) and depression (Fig. 5c) (as measured using the HADS).

#### Working life

**Fig. 6:**
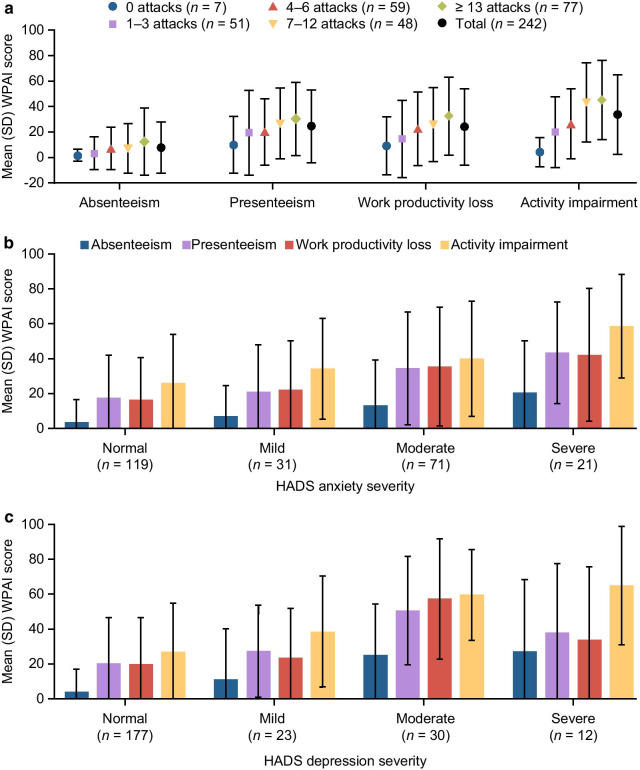
Mean WPAI scores. Scores are shown by the number of HAE attacks in the previous 6 months (**a**) and by HADS anxiety (**b**) and depression (**c**) subscales. *HADS* Hospital Anxiety and Depression Scale, *HAE* hereditary angioedema, *WPAI* Work Productivity and Impairment questionnaire

Impairment in working over the past week, in this case specifically due to HAE, was measured using the Work Productivity and Impairment questionnaire (WPAI) [18]. On average, patients reported being absent from work for 7.87% of the prior week (i.e. for a 40-h working week, the patient missed approximately 3 h of work). The mean (SD) percentage impairment measured by the WPAI was 24.59% (28.65) for presenteeism, 24.18% (30.03) for work productivity loss, and 33.88% (31.20) for activity impairment. Scores were lowest for patients in Germany and highest for patients in Spain (see Additional File 1: Table S9). Furthermore, scores for all domains were higher with a greater number of attacks (Fig. 6a) and as the severity of anxiety increased (Fig. 6b), whereas the effect of depression on impairment was greatest in patients with moderate depression (as measured using the HADS) (Fig. 6c).

## Discussion

This study recruited the broadest international sample of patients with HAE from Europe, Australia, and Canada for a patient survey to date. Given the rarity of HAE, the sample size in this study was large and provided an expansive exploration of the burden of illness of HAE across a large geographical area. It is also the first known evaluation of quality of life in patients from Australia, Switzerland, and Austria. The findings in this survey are in agreement with those reported from the related US patient survey [19], and with other evaluations of disease characteristics, burden, and unmet needs in the management of HAE in Europe and Canada [4–8, 20], confirming that issues faced by patients with HAE are not specific to a geographic region.

Patients reported an average of 25 HAE attacks in the past 12 months, and in almost half of patients, the most recent attack occurred within the last week. The majority of attack symptoms were moderate or severe, and approximately 42% of attacks lasted approximately 2 days or longer, indicating that disease activity remained high even after it was properly diagnosed and treatment was initiated. Notably, diagnosis was delayed by a mean of approximately 10 years across all countries after the first onset of symptoms, indicating a need for increased physician awareness of HAE.

The majority of patients carried on-demand medication at all times in accordance with international guidelines [21], and most attacks were treated with on-demand medication. However, some patients reported that they did not carry any on-demand medication in case of an attack, including patients who were using LTP. This reflects a gap in patient education, and patients should be made aware that breakthrough attacks may still occur during LTP use [22]. This could also result from limitations on the number of spare doses permitted by the patient’s health care system, leading patients to use their supply sparingly and only for more severe attacks [23].

Over half of patients were using LTP to control their attacks. LTP was generally effective, because many patients who used LTP reported few attacks compared with a high proportion of patients who reported many attacks and did not use LTP. Androgens were being used for LTP by over one-third of patients, despite well-documented side effects such as weight gain, virilization, menstrual abnormalities, headaches, and mood changes [24, 25], and tranexamic acid was used by almost 20% of patients, although its efficacy is not firmly established [26, 27]. Caution is recommended in deciding the appropriateness of androgens for LTP for individual patients [25], and guidelines do not support first-line use of tranexamic acid [9, 21]; thus, reasons for their continued use in HAE would be of interest to explore. In particular, the impact of convenience and dose frequency on adherence to oral versus intravenous treatments should be considered.

Interestingly, this survey also found that 20% of patients had used LTP in the past but discontinued. The proportion of past users was greater among patients who reported a high number of attacks in the past 6 months than those with few attacks. Sixteen patients (6.6%) reported using androgens as their most recent LTP, and of these, 3 (18.8%) discontinued based on physician recommendation. Given that almost half of patients reported in the AECT that they were “bothered” by the unpredictability of HAE attacks, and that scores were highest for the fears/shame domain of the AE-QoL (which includes a question about fear of the sudden onset of an attack), safe and effective LTP for prevention of attacks could be of significant benefit. Lower disease activity would also reduce the socioeconomic burden of HAE by decreasing the need for health care resource utilization and increasing work productivity.

Attack frequency was a major determinant of disease burden because quality of life, work productivity, and perception of disease control all worsened as attack frequency increased. Conversely, attack frequency did not affect the severity of anxiety and depression as measured by the HADS, the level of mental health impairment as measured by the SF-12v2, or fears/shame scores as measured by the AE-QoL. Anxiety and depression were, however, important factors contributing to the burden of HAE because patients with more severe anxiety or depression were more likely to show negative outcomes on all the other instruments administered. In particular, attack frequency, along with anxiety and depression, were drivers of poor disease control, as shown by the results from the AECT. Together, these data suggest that because of HAE, patients suffered from impaired mental health, particularly anxiety and depression, regardless of the severity of their disease as measured by the number of attacks. Even low HAE disease activity posed a burden, due to the perceived constant threat of an attack.

The unpredictable nature of HAE attacks may have been an important contributor to this mental health impairment [6], which in turn impacted other aspects of daily life. When asked about comorbidities, 26% of patients self-reported having anxiety, and 17% self-reported having depression. However, on the basis of the HADS subscale scores, 38% were classified as having moderate to severe anxiety and 17% were classified as having moderate to severe depression. This indicates that comorbid anxiety may have been underdiagnosed among patients with HAE.

Despite the clear burden of HAE, patients reported general satisfaction with their current HAE treatment: 73.2% were “satisfied,” “very satisfied,” or “extremely satisfied,” whereas 9.5% were “dissatisfied,” “very dissatisfied,” or “extremely dissatisfied.” Even among patients with ≥ 13 attacks in the past 6 months, 29.9% reported being satisfied to some degree. This response could have resulted from a sense of complacency, especially if resources were limited or other options were not readily available. Furthermore, patients may not have been aware of other treatments that are more tolerable and effective. This apparent discrepancy underlines the value of QoL assessment using validated tools for the management of HAE.

The survey also revealed gaps in patients’ understanding of HAE, as nearly one-third of patients that were screened out of the survey were unaware of which type of HAE they had. Furthermore, there remained some lack of adherence to international treatment guidelines; for example, approximately 5% of patients used tranexamic acid for the acute treatment of their most recent attack, even though guidelines state that such use is not recommended [9, 21].

### Study limitations

All survey responses were self-reported, with no third-party confirmation, and recall bias could lead to a misrepresentation of symptom experience. As there were few patients from Austria, Germany, and Switzerland who participated in the survey, the results from these countries should be interpreted with caution when compared with the results from other countries. There were few options for patients to provide free responses to survey questions and thus it is possible that granularity was lost from these responses. Although the demographic characteristics of the study population were varied, recruitment may have been biased for or against certain groups. For example, patients may have had more severe HAE because recruitment was conducted through patient advocacy organizations (PAOs), and patients with more severe disease may be more likely to turn to PAOs for support. In addition, online administration limits participation for those without internet access or those who are less comfortable using technology, such as older patients. Future studies would benefit from the inclusion of patients from more countries to further evaluate geographical differences, as well as confirmation of data through patient medical histories.

## Conclusions

Despite advances in HAE diagnostic strategies and the availability of more HAE treatment options, findings from this patient survey showed that many patients experienced frequent attacks of moderate to severe intensity, even with use of LTP. HAE imposed a substantial burden on patients in the countries included in this survey, including high levels of anxiety and depression and productivity impairment, even when patients were not having an attack. The findings from this study reflect the continuing need for improvements in care for patients with HAE. Keys to achieving this goal include better adherence to international guidelines, increased education for patients regarding compliance with and proper use of therapy, and more access to efficacious treatments.

## Methods

This was a noninterventional, cross-sectional, web-based survey of patients with a self-reported diagnosis of HAE. The survey was conducted in Australia, Austria, Canada, France, Germany, Spain, Switzerland, and the United Kingdom. Patients were recruited through PAOs by member organizations of HAE International, an international umbrella organization for HAE patient groups. Local PAOs recruited patients with HAE within each of their respective countries using telephone, email, website advertisements, and social media postings. Patients contacted the PAO, which then provided a brief overview of the study and a link to the survey. The survey was available in each country’s target language. The survey began with screening questions, and patients who passed the screening were required to give consent through the web link before completing the rest of the survey. Patients were compensated with 30 USD by the PAO.

The survey targeted enrollment of 10–90 patients per participating country, as estimated by local PAOs. Patients were ≥ 18 years of age with HAE type 1 or 2, had experienced ≥ 1 HAE attack or instance of prodromal symptoms within the last year, had received HAE medication within the last 2 years to treat an attack, were able to provide consent, and were fluent in the target language. Patients with HAE with normal C1-INH were excluded.

The survey included questions on patients’ medical history of HAE, including time since diagnosis, comorbidities, their most recent angioedema attack, current and past treatments (including use of prophylaxis), and treatment satisfaction. The impact of HAE on health-related quality of life was measured using the AE-QoL [17] and SF-12v2 questionnaires [11, 12]. Four domains (function, fatigue/mood, fears/shame, and nutrition) and a total score were assessed in the AE-QoL; the scoring system ranges from 0 to 100, with higher scores indicating higher levels of impairment and lower quality of life. In the SF-12v2, higher scores indicate better functioning, and the physical and mental health composite scores are normalized to a mean (SD) score of 50 (10) for the general population on both scales. Perceived control of the disease was measured using the AECT [15]. Patients were asked how often they had angioedema, how their quality of life was affected by angioedema, how much the unpredictability of angioedema bothered them, and how well their angioedema had been controlled by therapy. Scores for the responses in the AECT range from 0 to 16, with higher scores indicating better disease control (≤ 9 poorly controlled; ≥ 10 well controlled). The ability to work and participate in regular activities was measured using the WPAI [18]; WPAI scores indicate the percentage of time the patient missed work or was less productive owing to HAE-related complications. Four domains were assessed: absenteeism, presenteeism, work productivity loss, and activity impairment. Mental health was measured using the HADS [13, 14], where total scores range from 0 to 42, with subscale scores ranging from 0 to 21 (0–7 is considered normal, 8–10 mild, 11–14 moderate, and 15–21 high for levels of anxiety or depression). General health and sociodemographic information were also collected.

All data were summarized using descriptive statistics. Subgroups that were analyzed included country, HAE type, number of angioedema attacks in the past 6 months (0 attacks, 1–3 attacks, 4–6 attacks, 7–12 attacks, ≥ 13 attacks), and HADS subscale scores for anxiety and depression (for each subscale, including normal, mild, moderate, and severe).

## Supplementary information

**Additional file 1: Supplementary information. Table S1. **Delay in diagnosis by country. **Table S2.**Number of HAE attacks in the past 6 months by country. **Table S3. **Number of patients visiting health care providers by country. **Table S4. **Number of emergency room visits, urgent care center visits, and hospitalizations by country. **Table S5. **SF-12v2 scores by country. **Table S6. **HADS scores by country. **Table S7. **AECT scores by country. **Table S8. **AE-QoL scores by country. **Table S9.** WPAI scores by country. **Figure S1. **Patient flow. **Figure S2. **Comorbities. **Figure S3. **Current LTP use by country. **Figure S4. **STP use by country. **Figure S5. **Mean SF-12v2 physical and mental health composite scores. **Figure S6.** AECT scores by number of attacks in the previous 6 months

## Data Availability

The data generated during the current study are proprietary and not publicly available, but data are available from the corresponding author on reasonable request and with permission of Takeda Pharmaceutical Company Limited.

## Abbreviations

AECT: Angioedema Control Test
AE-QoL: Angioedema Quality of Life Questionnaire
C1-INH: C1 inhibitor
HADS: Hospital Anxiety and Depression Scale
HAE: Hereditary angioedema
LTP: Long-term prophylaxis
PAO: Patient advocacy organization
SF-12v2: 12-Item Short-Form Health Survey
STP: Short-term prophylaxis
WPAI: Work Productivity and Impairment questionnaire
