# Supplementary material for: Clinical characteristics and burden of illness in patients with hereditary angioedema: findings from a multinational patient survey
Source: Orphanet J Rare Dis. 2021 Feb 18;16:94. doi: 10.1186/s13023-021-01717-4 (PMC7893968; doi:10.1186/s13023-021-01717-4)

Supplementary information

AU = Australia

AT = Austria

CA = Canada

FR = France

DE = Germany

ES = Spain

CH = Switzerland

UK = United Kingdom

Table S1. Delay in diagnosis by country

Age, years	Total (N = 242)	AU (n = 28)	AT (n = 13)	CA (n = 32)	FR (n = 58)	DE (n = 7)	ES (n = 39)	CH (n = 8)	UK (n = 57)
**At onset**									
Mean (SD)	11.54 (8.90)	9.68 (9.20)	11.92 (6.41)	13.72 (13.66)	12.81 (9.79)	8.57 (5.59)	11.90 (6.94)	10.88 (7.53)	10.07 (6.17)
Median (range)	10.00 (0–58)	7.50 (2–50)	11.00 (4–21)	12.00 (1–58)	12.00 (0–56)	8.00 (2–16)	12.00 (1–27)	8.00 (4–25)	8.00 (0–30)
**At diagnosis**									
Mean (SD)	20.81 (13.16)	19.07 (15.21)	17.69 (10.25)	22.16 (15.28)	22.78 (13.74)	12.71 (7.65)	22.90 (12.81)	22.38 (7.27)	18.95 (11.91)
Median (range)	19.00 (0–59)	16.00 (0–50)	21.00 (4–34)	20.50 (2–59	21.00 (0–57)	16.00 (1–21)	23.00 (1–55)	24.50 (6–30)	18.00 (0–50)

Table S2. Number of HAE attacks in the previous 6 months by country

Number of attacks	Total (N = 242)	AU (n = 28)	AT (n = 13)	CA (n = 32)	FR (n = 58)	DE (n = 7)	ES (n = 39)	CH (n = 8)	UK (n = 57)
Mean (SD)	12.51 (14.10)	14.61 (16.50)	13.00 (7.19)	12.56 (11.67)	7.10 (8.88)	15.14 (6.49)	9.72 (13.32)	18.38 (19.00)	17.60 (17.83)
Median (range)	7.00 (0–90.00)	7.00 (0–60.00)	15.00 (3.00–25.00)	9.00 (1.00–40.00)	4.50 (0–52.00)	15.00 (2.00–22.00)	6.00 (0–78.00)	14.50 (4.00–61.00)	12.00 (1.00–90.00)

Table S3. Number of patients visiting health care providers by country

Provider, no. of patients (%)	Total (*N* = 242)	AU (*n* = 28)	AT (*n* = 13)	CA (*n* = 32)	FR (*n* = 58)	DE (*n* = 7)	ES (*n* = 39)	CH (*n* = 8)	UK (*n* = 57)
Allergist/immunologist	156 (64.5)	26 (92.9)	1 (7.7)	24 (75.0)	11 (19.0)	1 (14.3)	38 (97.4)	7 (87.5)	54 (94.7)
GP/internist	97 (40.1)	16 (57.1)	4 (30.8)	6 (18.8)	40 (69.0)	1 (14.3)	13 (33.3)	3 (37.5)	14 (24.6)
PA/nurse practitioner	23 (9.5)	2 (7.1)	0	5 (15.6)	6 (10.3)	0	8 (20.5)	0	2 (3.5)
Hematologist	18 (7.4)	0	0	6 (18.8)	4 (6.9)	3 (42.9)	0	4 (50.0)	1 (1.8)
Dermatologist	17 (7.0)	0	12 (92.3)	2 (6.3)	3 (5.2)	0	0	0	0
OB/GYN	11 (4.5)	1 (3.6)	0	0	6 (10.3)	0	2 (5.1)	0	2 (3.5)
Rheumatologist	4 (1.7)	0	0	1 (3.1)	1 (1.7)	0	1 (2.6)	0	1 (1.8)
Gastroenterologist	3 (1.2)	0	0	0	1 (1.7)	0	0	1 (12.5)	1 (1.8)

GP General practitioner, *PA* physician assistant, *OB/GYN* obstetrician-gynecologist

Table S4. Number of emergency room visits, urgent care center visits, and hospitalizations by country

Facility, mean no. of visits (SD)	Total (*N* = 242)	AU (*n* = 28)	AT (*n* = 13)	CA (*n* = 32)	FR (*n* = 58)	DE (*n* = 7)	ES (*n* = 39)	CH (*n* = 8)	UK (*n* = 57)
Emergency room/A&E visit	1.94 (6.02)	3.21 (5.61)	7.38 (17.12)	1.19 (2.47)	0.59 (1.53)	0.43 (1.13)	2.77 (4.32)	0.75 (1.75)	1.67 (6.93)
Urgent care center visit	1.03 (5.42)	1.25 (2.43)	0.08 (0.28)	0.09 (0.39)	0.09 (0.34)	0.14 (0.38)	3.95 (8.94)	0.75 (1.75)	0.30 (0.87)
Hospitalization	1.04 (5.10)	2.21 (11.33)	6.54 (17.49)	0.31 (0.74)	0.40 (1.39)	0.14 (0.38)	0.74 (2.47)	0.13 (0.35)	1.18 (5.38)

A&E accident and emergency

Table S5. SF-12v2 scores by country

SF-12v2 score, mean (SD)	AU (*n* = 28)	AT (*n* = 13)	CA (*n* = 32)	FR (*n* = 58)	DE (*n* = 7)	ES (*n* = 39)	CH (*n* = 8)	UK (*n* = 57)
Physical function	51.05 (9.70)	56.03 (2.97)	50.34 (9.78)	53.03 (8.65)	54.99 (5.97)	47.13 (11.31)	54.29 (4.09)	47.83 (10.17)
Role physical	47.29 (9.54)	47.02 (7.41)	47.98 (10.61)	47.48 (9.99)	51.05 (8.02)	42.16 (10.98)	47.59 (5.86)	43.91 (9.47)
Bodily pain	47.79 (9.49)	48.18 (9.86)	46.86 (11.55)	47.61 (9.82)	50.16 (7.18)	42.09 (13.92)	48.64 (7.93)	42.29 (12.05)
General health	49.10 (7.73)	49.86 (9.14)	48.85 (9.90)	49.10 (8.49)	50.28 (8.51)	42.68 (10.82)	51.22 (6.06)	44.82 (10.54)
Vitality	48.18 (8.57)	52.32 (9.49)	48.92 (9.03)	49.49 (9.75)	50.80 (9.84)	48.78 (10.63)	55.24 (4.76)	44.65 (11.01)
Social functioning	47.25 (10.83)	46.21 (8.83)	45.76 (11.22)	46.12 (9.55)	49.57 (4.52)	39.08 (10.94)	46.92 (8.57)	41.72 (10.79)
Role emotional	42.79 (12.61)	47.10 (12.07)	45.99 (11.52)	45.50 (12.46)	42.38 (8.58)	33.29 (14.05)	49.75 (7.51)	37.48 (13.29)
Mental health	45.07 (9.56)	50.27 (9.91)	48.86 (8.16)	48.64 (9.90)	48.03 (11.79)	41.50 (9.78)	49.21 (7.80)	41.81 (9.13)
Physical Health Composite	51.01 (8.21)	51.49 (4.93)	49.18 (10.92)	50.60 (7.42)	54.54 (6.65)	46.82 (11.15)	51.24 (4.15)	47.32 (10.24)
Mental Health Composite	43.63 (12.06)	47.56 (11.60)	46.80 (8.85)	46.08 (10.52)	44.50 (9.35)	37.91 (11.22)	49.33 (6.67)	39.18 (11.19)

SF-12v2 12-Item Short-Form Health Survey

Table S6. HADS scores by country

HADS score, mean (SD)	Total (*N* = 242)	AU (*n* = 28)	AT (*n* = 13)	CA (*n* = 32)	FR (*n* = 58)	DE (*n* = 7)	ES (*n* = 39)	CH (*n* = 8)	UK (*n* = 57)
Anxiety	8.05 (4.54)	8.82 (4.88)	4.15 (3.87)	6.75 (3.84)	6.57 (3.63)	6.29 (2.63)	10.23 (4.92)	5.75 (2.66)	9.82 (4.41)
Depression	5.39 (4.36)	5.36 (4.37)	3.62 (3.91)	4.34 (3.45)	4.16 (2.98)	2.43 (3.10)	7.59 (5.11)	2.38 (1.06)	6.93 (4.85)
Total	13.43 (8.17)	14.18 (8.52)	7.77 (7.63)	11.09 (6.33)	10.72 (5.70)	8.71 (5.22)	17.82 (9.59)	8.13 (3.27)	16.75 (8.22)

HADS Hospital Anxiety and Depression Scale

Table S7. AECT scores by country

AECT score	Total (N = 242)	AU (n = 28)	AT (n = 13)	CA (n = 32)	FR (n = 58)	DE (n = 7)	ES (n = 39)	CH (n = 8)	UK (n = 57)
Mean (SD)	8.00 (3.44)	8.86 (4.12)	6.23 (1.92)	9.06 (2.72)	9.69 (3.44)	7.00 (2.31)	6.77 (3.25)	6.63 (1.51)	6.82 (3.19)
Median (range)	8.00 (0–16.00)	9.00 (2.00–16.00)	6.00 (3.00–10.00)	9.00 (1.00–16.00)	10.00 (2.00–16.00)	6.00 (4.00–11.00)	6.00 (0–14.00)	6.50 (4.00–9.00)	7.00 (0–13.00)

AECT Angioedema Control Test

Table S8. AE-QoL scores by country

AE-QoL score, mean (SD)	Total (*N* = 242)	AU (*n* = 28)	AT (*n* = 13)	CA (*n* = 32)	FR (*n* = 58)	DE (*n* = 7)	ES (*n* = 39)	CH (*n* = 8)	UK (*n* = 57)
Functioning	42.46 (27.39)	36.38 (28.47)	50.48 (22.02)	34.18 (24.95)	34.91 (30.80)	30.36 (17.09)	53.53 (24.95)	36.72 (27.02)	50.66 (23.88)
Fatigue/mood	46.24 (24.95)	48.21 (25.50)	39.62 (31.39)	44.69 (20.04)	35.17 (21.07)	28.57 (21.74)	55.77 (23.44)	36.25 (16.85)	55.96 (25.87)
Fears/shame	54.68 (24.67)	51.19 (26.30)	29.49 (20.59)	46.35 (19.74)	54.17 (24.51)	29.17 (19.69)	69.44 (19.03)	35.94 (21.59)	63.01 (21.78)
Nutrition	36.16 (28.62)	34.82 (25.08)	25.00 (21.65)	26.17 (21.38)	31.03 (28.22)	14.29 (19.67)	42.95 (32.16)	23.44 (33.70)	50.00 (27.35)
Total	47.14 (20.69)	44.91 (23.43	36.88 (20.27)	40.63 (15.52)	41.33 (19.98)	27.52 (13.01)	58.56 (17.34)	34.74 (16.32)	56.50 (18.91)

AE-QoL Angioedema Quality of Life questionnaire

Table S9. WPAI scores by country

WPAI score, mean (SD)	Total (*N* = 242)	AU (*n* = 28)	AT (*n* = 13)	CA (*n* = 32)	FR (*n* = 58)	DE (*n* = 7)	ES (*n* = 39)	CH (*n* = 8)	UK (*n* = 57)
Absenteeism	7.87 (20.01)	5.08 (15.57)	3.59 (9.04)	4.55 (16.11)	7.27 (20.61)	0	17.25 (29.83)	0	8.60 (19.06)
Presenteeism	24.59 (28.65)	25.56 (32.94)	18.89 (17.64)	20.00 (31.12)	19.23 (27.41)	10.00 (10.00)	33.33 (35.68)	12.00 (21.68)	29.76 (25.71)
Work productivity loss	24.18 (30.03)	27.22 (34.95)	21.51 (19.86)	19.17 (30.57)	15.47 (26.67)	10.00 (10.00)	32.86 (36.94)	10.00 (20.00)	32.18 (28.54)
Activity impairment	33.88 (31.20)	33.21 (30.07)	35.38 (27.57)	25.31 (29.29)	23.97 (31.06)	11.43 (10.69)	50.77 (34.52)	20.00 (26.19)	41.93 (26.95)

WPAI Work Productivity and Impairment questionnaire

Figure S1. Patient flow

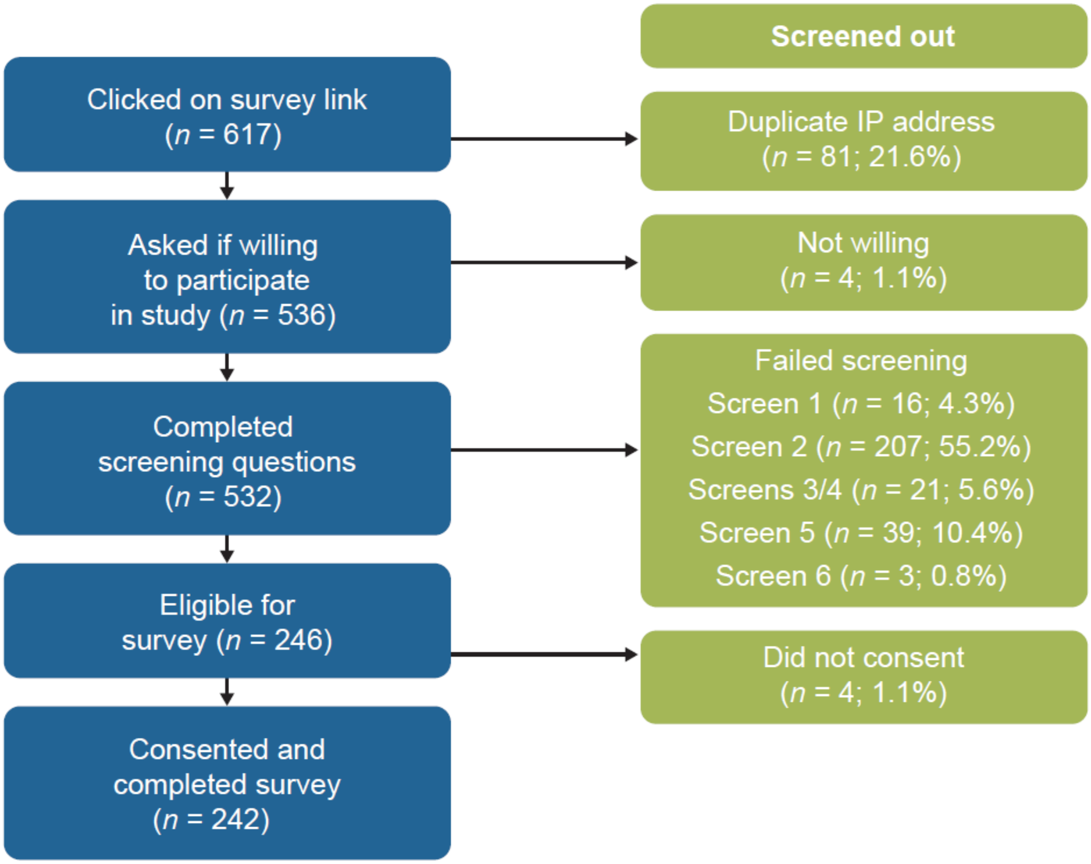

Percentages for patients screened out are calculated from a total of 375 patients excluded. Screen 1: "*Has a healthcare professional told you that you have hereditary angioedema, or HAE*?" Screen 2: *"What type of HAE do you have?*" Screen 3: *"In the past year (12 months), how many angioedema attacks did you have? Please include any attacks in which you experienced swelling, abdominal pain, nausea, vomiting, diarrhea, or other symptoms related to your attack."* Screen 4: *"In the past year (12 months), how often did you have signs or symptoms that suggest that an attack was coming WITHOUT having an angioedema attack?"* Screen 5: *"Have you taken a prescription medication to treat or prevent an angioedema attack in the past 2 years?"* Screen 6: *"What is your current age?"*

Figure S2. Comorbidities

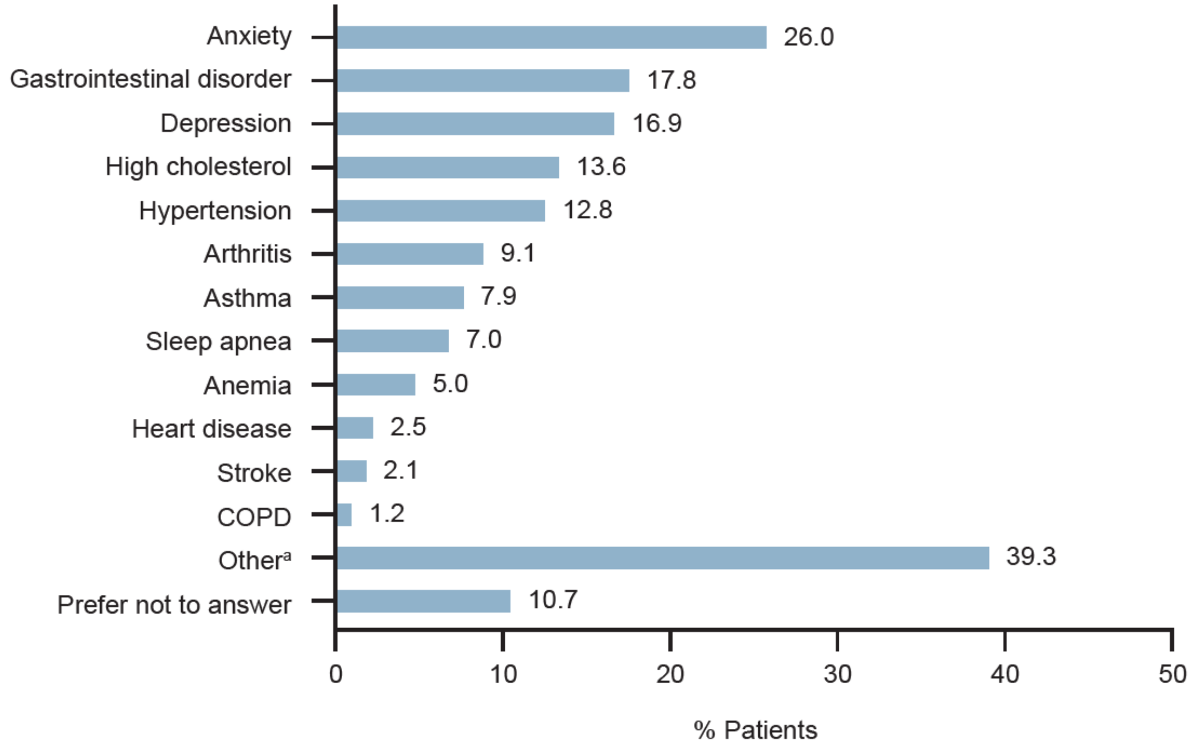

*A total of 50 "other" comorbidities were reported by patients as free text, including thyroid conditions (n=7, 2.9%), arthritis (n=3, 1.2%), and diabetes (n=3, 1.2%). *COPD* chronic obstructive pulmonary disease.

Figure S3. Current LTP use by country

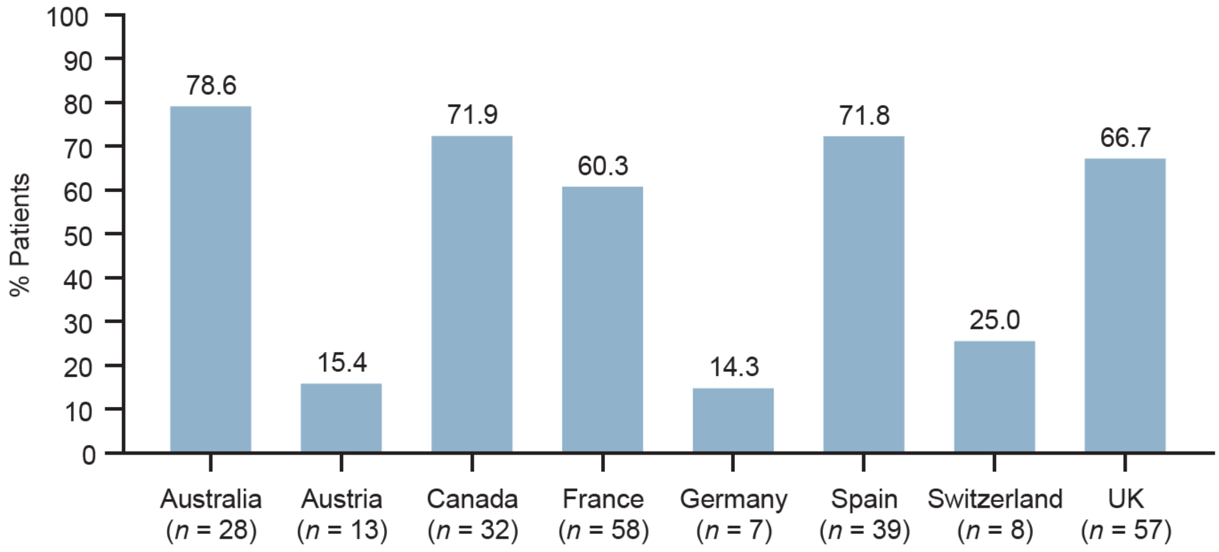

Figure S4. STP use by country

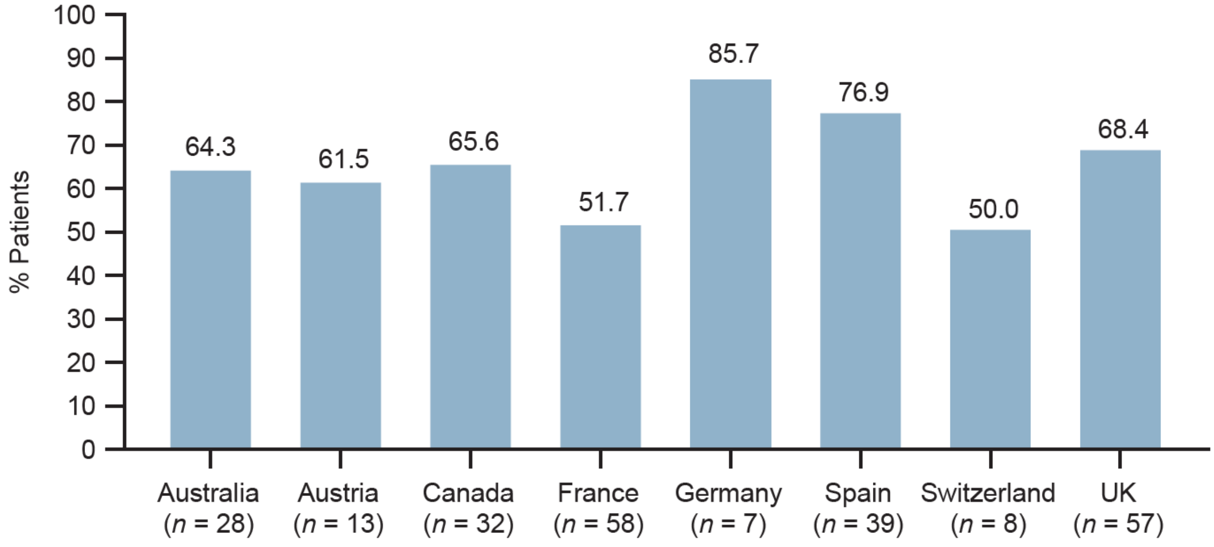

Figure S5. Mean SF-12v2 physical and mental health composite scores

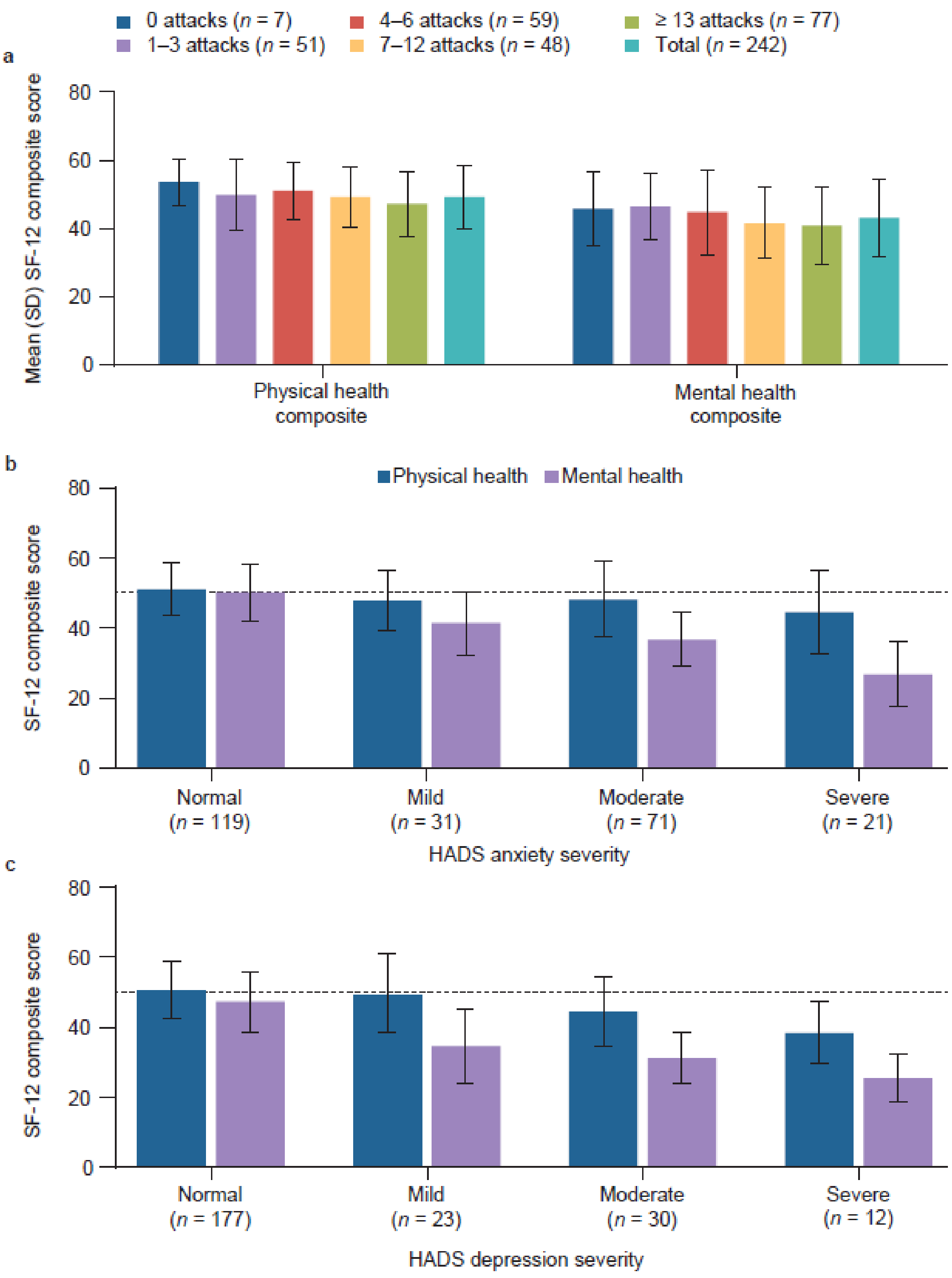

Scores are shown by number of HAE attacks in the previous 6 months (a) and by HADS anxiety (b) and depression (c) subscales. The dotted line indicates a mean score of 50 which represents

the general population. *HADS* Hospital Anxiety and Depression Scale, *HAE* hereditary angioedema, *SF-12v2* 12-Item Short-Form Health Survey

Figure S6. AECT scores by number of attacks in the previous 6 months

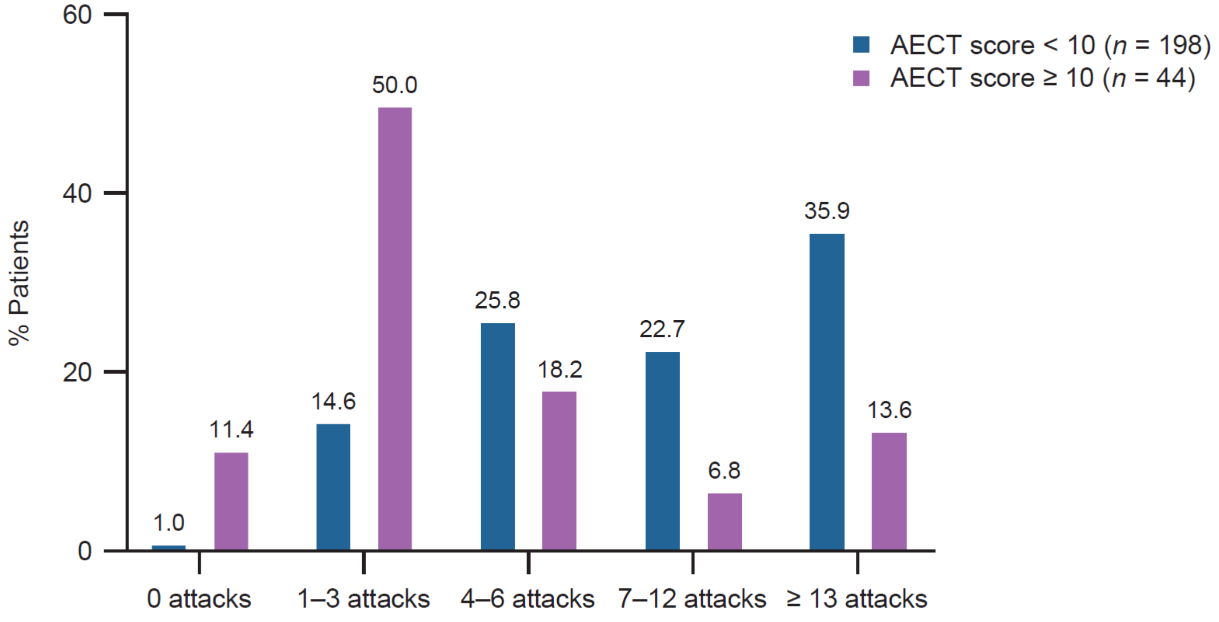

Supplement: Supplementary file 1 — Additional file 1: Supplementary information. Table S1. Delay in diagnosis by country. Table S2.Number of HAE attacks in the past 6 months by country. Table S3. Number of patients visiting health care providers by country. Table S4. Number of emergency room visits, urgent care center visits, and hospitalizations by country. Table S5. SF-12v2 scores by country. Table S6. HADS scores by country. Table S7. AECT scores by country. Table S8. AE-QoL scores by country. Table S9. WPAI scores by country. Figure S1. Patient flow. Figure S2. Comorbities. Figure S3. Current LTP use by country. Figure S4. STP use by country. Figure S5. Mean SF-12v2 physical and mental health composite scores. Figure S6. AECT scores by number of attacks in the previous 6 months [file 13023_2021_1717_MOESM1_ESM.pdf]
